# Additive Timber Manufacturing: A Novel, Wood-Based Filament and Its Additive Robotic Fabrication Techniques for Large-Scale, Material-Efficient Construction

**DOI:** 10.1089/3dp.2020.0356

**Published:** 2022-06-09

**Authors:** Philipp Eversmann, Julian Ochs, Jannis Heise, Zuardin Akbar, Stefan Böhm

**Affiliations:** ^1^ Department of Experimental and Digital Design and Construction and Universität Kassel, Kassel, Germany.; ^2^ Department of Separating and Joining Manufacturing Processes, Universität Kassel, Kassel, Germany.

**Keywords:** timber construction, robotic fabrication, sustainability, additive manufacturing, 3d printing, bio-printing

## Abstract

Additive manufacturing (AM), as resource-efficient fabrication processes, could also be used in the dimensions of the construction industry, as a variety of experimental projects using concrete and steel demonstrate. In timber construction, currently few additive technologies have been developed having the potential to be used in large scale. Currently known AM processes use wood in pulverized form, losing its inherent structural and mechanical properties. This research proposes a new material that maintains a complete wood structure with continuous and strong fibers, and that can be fabricated from fast-growing locally harvested plants. We describe the material technology to create a solid and continuous filament made of willow twigs and investigate binding and robotic AM methods for flat, curved, lamination, and hollow layering geometric typologies. The resulting willow filament and composite material are characterized for structural capacity and fabrication constraints. We discuss our technology in comparison with veneer-based lamination, existing wood filament printing, and fiber-based AM in terms of fabrication, material capacity, and sustainability. We conclude by showing possible applications in the construction industry and future research possibilities.


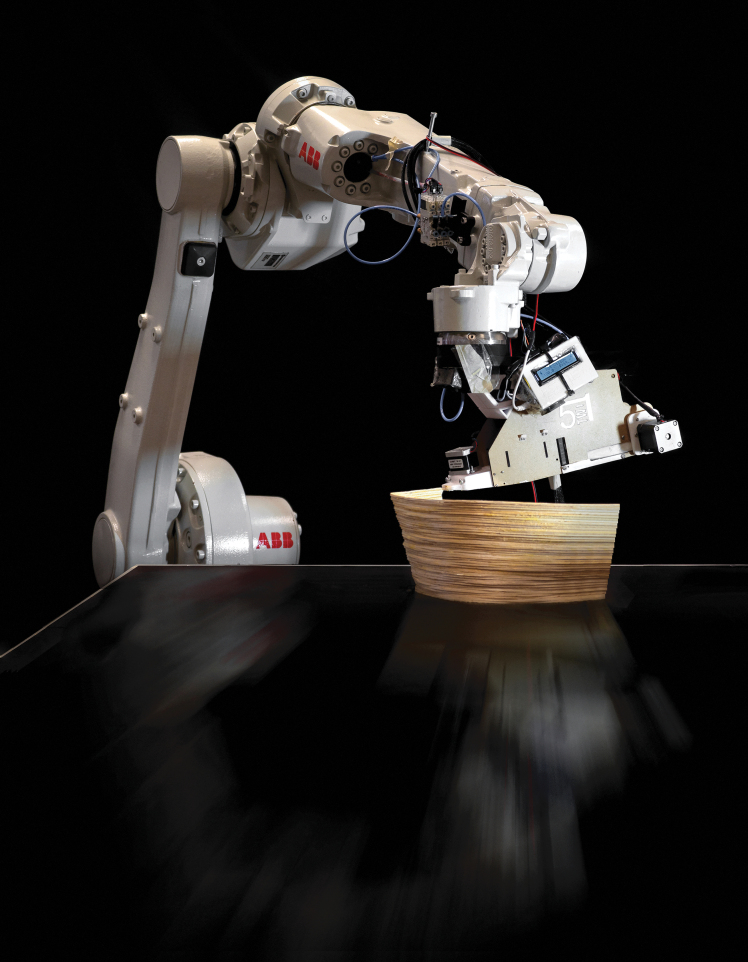


## Introduction

There is a great demand for new construction, which has increased the volume of orders in the construction industry by 1.5-times over the last 10 years in Germany.^1^ According to forecasts, the volume of materials used will continue to double by 2060.^2^ Material-saving and fast design, and manufacturing and assembly processes should therefore be used.

With the current building techniques and available resources, this is hardly feasible and, above all, in relation to the CO_2_ balance, especially for concrete construction. Due to the positive ecological balance, and already established digital workflows, this has led to a rapid increase of timber construction. At present, however, little attention is paid to resource-saving construction methods, and consequently, enormous masses of cross-laminated timber and stacked timber are used.^3^ Here, additive manufacturing (AM) processes could be very promising and resource-efficient construction processes, as a variety of pilot projects using concrete and steel demonstrate.^4,5^ The following sections give an overview on veneer-based lamination, and wood filament- and fiber-based AM technologies, which form a background for this research.

### Veneer-based lamination

Molded wood is a manufacturing technique in which several veneer layers are processed in a press under heat and pressure to form two- or three-dimensional shapes. Patents exist for laying veneer layers automatically to fabricate laminated or cross-laminated plates.^6–8^ These techniques can also be seen as a predecessor of an AM technique, since an addition of layers of material are used to create a final shape, even though this is not done in a continuous process.

The furniture designer Michael Thonet had been experimenting with the gluing of veneer layers since 1830.^9^ The first patented shaped wood process, however, was developed by Isaak Cole in 1874.^10^ It was not until the 1930s that different products were introduced to the market, followed 10 years later by the famous plywood furniture collection by Charles and Ray Eames and in 1952 by Arne Jacobsen.^11,12^ Apart from the design classics from these years, shaped wood is particularly suitable for interior design, seating, bed slats, skateboards, and vehicle construction due to its high strength and low weight. The size of components made of molded wood is limited by the existing presses, so molded wood processes are mainly used for smaller components as furniture components or construction plates.

In building construction, structural components made from hardwood veneers such as beech have been developed for high-performance applications as beams and plate structures.^13^ As adhesives, melamine urea formaldehyde (UF) resins, phenol formaldehyde resins, and polyurethane adhesives are used. Veneer-based products have with around 3% a higher adhesive/volume ratio than cross-laminated timber (around 2.1%). Despite the use of adhesive, they keep a largely negative CO_2_ emission (Veneer-based: −424 kg CO_2_-eq, CLT: −524 kg CO_2_-eq).^14^

### Filament-based AM with wood

3D printing filaments with wood content are already available in the market for fused deposition modeling (FDM). Polylactic acid (PLA) filament enriched with wood flour (5 wt%) was developed by Tao *et al*.^15^ Similar filaments with a higher wood content, namely 10%, 20%, and 30%, were produced by double-shear corner extrusion.^16^ A higher wood content led to the reduction of mechanical properties, since the presence of wood flour alters the microstructure of the matrix, resulting in a reduction in the interfacial compatibility. Superior mechanical properties were developed by Pitt *et al.*,^17^ which were mainly attributed to improved interlayer interaction through filament overlap, uniform dispersion of wood powder, densification of starting material paste, and fiber orientation. Filaments with up to 30 wt% surface-modified micro/nanocellulose PLA were prepared by Wang *et al.*^18^ Advanced fiber-reinforced polymers (including cellulosic nanofibers) were presented by Parandoush and Lin.^19^ 3D printing of wood flour, in combination with a binder of cellulose, nanocrystals, and xyloglucan, is also possible.^20^ The first attempts to print wood-based materials using liquid deposition modeling are described by Kariz *et al.*,^21^ using polyvinyl acetate (PVAc) and UF as binders (with up to 15–20 wt% wood powder). Rosenthal *et al.*^22^ were able to increase the wood content in experimental studies to 90%. Using the anisotropic and hygroscopic structures of the wood fiber direction, wood composites were developed at MIT's Self-Assembly Lab, which automatically deform when humidity changes.^23^ The TU Dresden is currently developing an AM process based on the extrusion of a paste-like mass of wood chips and binders in cooperation with the West Saxon University of Applied Sciences Zwickau.^24^

### Fiber-based AM with biomaterials

Flax, hemp, and ramie are among the natural fibers with the highest specific modulus of elasticity and tensile strength. However, a great deal of variability in the literature should be noted. In general, geography plays an important role in the selection of fibers in terms of availability.^25,26^ Applications of biofiber composites in the automotive industry have been investigated.^27^ Recent studies show FDM with natural fibers, and with continuous fiber-reinforced biocomposites.^28,29^ This is achieved by separately feeding a filament and continuous fibers.^30^ Hinchfliffe *et al.*^31^ show that mechanical properties can be improved by prestressing the fibers. Electro-, dry-, and wet-spinning of cellulosic materials has made it possible to produce submicron fibers and ultrafine filaments with a strong oriented structure.^32^ The extraction of natural fibers for modern technical use achieves a fiber yield of around 25%.^33^ The ecological footprint of natural fibers depends on the country of cultivation and the associated transport routes, in addition to the complex manufacturing process. For example, the ecological footprint of the natural fibers hemp, flax, jute, and kenaf is between 350–975 kg CO_2_-eq per ton of fiber for the countries of origin Germany and India.^34^ For architectural production, various research projects are currently investigating new methods, as the robotic winding of mainly carbon and glass fibers to form lightweight structures.^35–39^ While impressive structural capacities can be reached with these materials, their ecological impact should be carefully considered, since the production of carbon fibers results in an estimated 30.000–80.000 kg CO_2_-eq per ton of fiber and in 1700–2500 kg CO_2_-eq per ton of fiber for glass fibers.^40,41^

As this investigation of the state-of-the-art shows, AM processes are still hardly used in timber construction, despite its high level of digitization and technical development. Currently known processes use wood in pulverized form or as bulk material on a wood basis, which, however, means that the material properties are largely lost.^42,43^ This research investigates the use of solid wood for AM. Thus, inherent wood properties, such as anisotropy, can be retained and specifically used in the application.

## Materials and Methods

The goal of this research is to develop a new AM method for large-scale timber components. For this purpose, a printing material is needed, which is at least as strong as timber used in construction, can be produced sustainably in large quantities, and can be manufactured continuously. The AM process should also be ideally considerably faster than traditional 3D printing to be efficient for large components, which means that it needs an extrusion and binding technology suitable for high-speed automation. In terms of the produced parts, the technique should allow for a large range of possible shapes with as much geometric freedom as possible. In this section, we therefore describe the material development, binding experiments, robotic AM, and structural testing.

### Material development

In the joint research project “FLIGNUM,” a continuous solid wood filament was developed. Although the original aim of the project was to produce a wooden yarn for use in industrial textile production, its application as a filament in AM was also investigated.

**FIG. 1.**
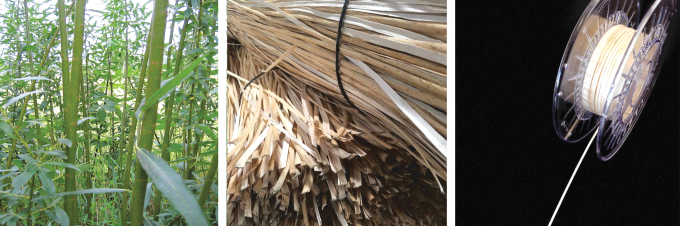
(*Left*) Willow twig plantation of “Salix Americana” genus: The twigs can be harvested in 1-year cycles. (*Middle*) The outer shell of the harvested twigs is split in multiple strips after harvesting. (*Right*) Continuous, solid willow filament rolled on a regular 3D printing spool.

This filament is produced in the following way: Annual willow rods of the genus “Salix Americana,” which can be grown regionally and sustainably as a shrub plant, are used as the raw material. Similar to bamboo, the outer surface is the strongest part of the material.^44^ After harvesting, this outer surface can be divided into several thin strips of 1.2–2.0 m. Since the cross section of the strips is not uniform (4.9–6.5 mm in width and 0.7–1.4 mm in thickness), the strips are then homogenized by micro-cutting. This involves trimming both sides and surfaces of the willow rods, with processes such as planing, milling, and sawing scaled down to these small dimensions. The homogenized strips are then adhesively joined to form a continuous filament. The raw material, the split willow rods, the homogenized strips, and the continuous wood filament can be seen in Figure 1.

**FIG. 2.**
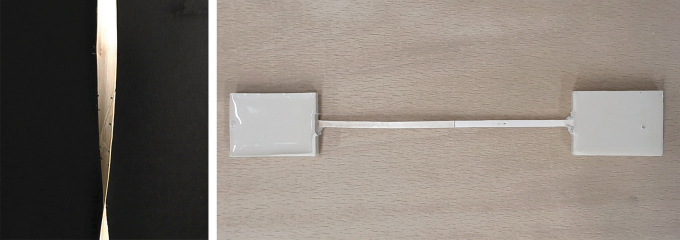
(*Left*) The filament joint with an angled cut to maximize the surface area for adhesives. (*Right*) Specimen ends were embedded in casting resin to prevent defects by clamping into the test machine.

For joining the willow strips, a variety of simple overlap joints up to detailed microjoining geometries were investigated. Since the research project is still ongoing, multiple joining geometries, adhesive systems, and microdosing possibilities are still being investigated. In general, traditional wood adhesives (e.g., PVAc or PUR) are well suited for joining. However, properties such as foaming or long binding times require longer cycle times or have an influence on the aesthetics of the joint. For this reason, hotmelt adhesives, light-activated adhesives, and dual-curing adhesive systems are also being considered in the project. One of the most promising joining geometries is an angular cut in the height of the profile, which at the same time maximizes the surface of the joint and allows a continuous profile of the filament (Fig. 2). The finished filament can then be wound onto regular 3D printing spools for AM.

### Binding experiments

The joining time limits the processing and application in terms of time and thus significantly determines its productivity and quality. To enable a high process speed in AM, a suitable joining technology was required. A distinction must be made between the production of the continuous filament from solid wood and the joining process during AM. Essential requirements are the possibility of a direct and precise quantity, the fast reach of a sufficiently strong initial strength, as well as the integrability into a robotic system with accompanying high repetitive accuracy. Furthermore, the joints must have a high visual quality and at the same time be able to withstand mechanical stress. The filament is also required to have a largely homogeneous flexibility over the entire length. For these reasons, experimental investigations of contact adhesives, hotmelt adhesives, and UV-curing adhesives or resin systems were explored, as deemed suitable for both applications. To produce continuous filaments, which is decoupled from AM, classic adhesives for wood processing, such as PUR or PVAc, could also be suitable. For the present work, bonding tests were made with a dispersion contact adhesive and an acrylic UV-resin. As samples, willow rails with different overlap lengths have been glued together. These tensile shear specimens are tested on a Zwick&Roll Z100 universal testing machine at a test speed of 10 mm/min with 10 test specimens for each test variant. A force drop of 80% is defined as a criterion for the end of the test. Due to the filigree structure of the single wooden rails, the specimens can cause predamage and thus result in an early failure, if they are too firmly clamped in the specimen holder. To prevent this effect, a specimen head is fixed at both ends of the specimens using a 2K-PUR rapid casting resin, shown in Figure 2. As mentioned above, bonding tests were carried out using a UV-light-curing acrylic resin. The aim of the tests was the experimental determination of the possibility to completely cure the resin even in the shadow areas. The experimental setup comprised a multispectral LED UV light source, two optical fibers, and a laboratory stand for fixing and aligning the light spots. The dispersion contact adhesive was processed according to the technical data sheet.

### Robotic AM: fabrication process, tool design, and experiments

A robotic AM process with continuous wood filament must meet a number of requirements for the widest possible range of applications in different industries, such as geometric freedom, reliability, speed, and precision. To meet all these requirements, a novel overall process was developed, which is described in this section. We performed various experiments to define the fabrication boundaries and demonstrate the geometric possibilities of our additive timber manufacturing technique. This was done for four geometric typologies: flat-panel fabrication, multilayer lamination, curved-surface fabrication, and hollow-core application.

**FIG. 3.**
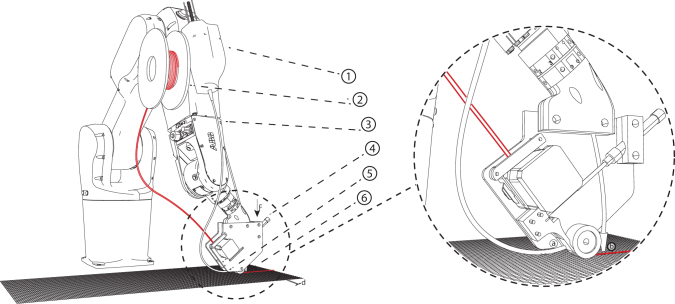
Robotic setup for planar surface additive timber manufacturing: syringe extruder for glue application (1), filament spool (2), robotic arm (3), pressure-applying end effector (4), glue-application nozzle (5 & a), and UV-curing light (6 & b).

For a fast and reliable AM process, our end effector had to perform several tasks as follows: loading, cutting, and guiding the filament, and adhesive application and activation (Fig. 3). A Nema-17 stepper motor was used for filament extrusion. Its necessary application pressure was achieved through a gear roller with a spring-loaded ball bearing. We used 3D-printed parts to guide the filament to a pneumatically actuated blade for cutting the filament to length, and an additional flexible guide component in front of the silicone roller to assure a precise application to the print bed. Different adhesives require specific application procedures. Contact adhesives can be precoated on the filament and activated by targeted pressure from the robot arm; UV adhesives can be applied directly in the end effector and activated via lateral exposure through liquid light guides from the UV source.

We developed our own open-source robot simulation software, Robot Components, which is capable of simulating different toolpath strategies for each typology.^45^ The software can also integrate several external axis types, linear and rotational, which are necessary for large-scale production and hollow-core application techniques.

For the fabrication of flat panels, we investigated a method similar to Automated Tape Laying, allowing an efficient application from filament spools.^46^ This was done with a setup of a six-axis robot together with the previously described end effector, enabling the precise production of flat, fabric-like panels or bodies. For the toolpath generation for flat surfaces, we used u,v-isocurves, constrained with the width of the filament with additional spacing for material tolerances.

**FIG. 4.**
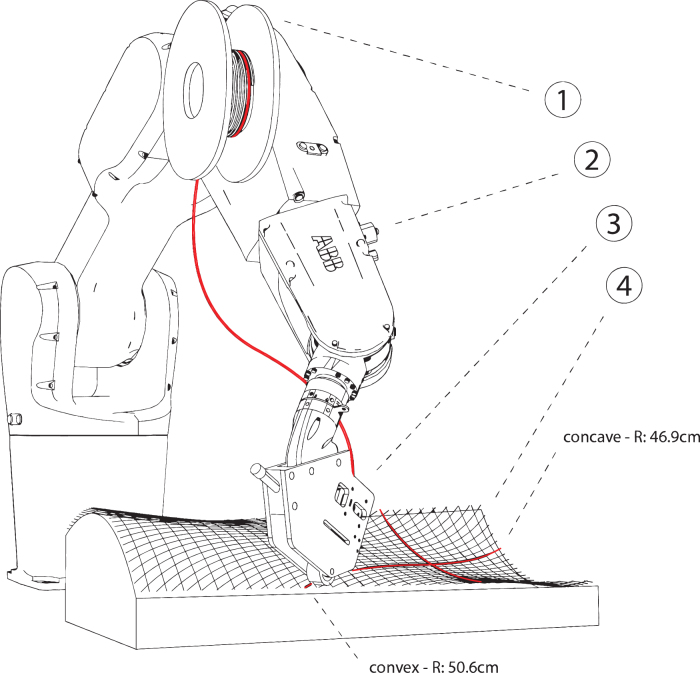
Robotic setup for curved surface additive timber manufacturing: end effector (3), on an industrial robot arm (2), with willow filament on a spool (1), on a supporting structure (4).

For the fabrication of curved surfaces, we investigated the use of used uncoated 5-axis milled foam molds as a base for material layup (Fig. 4). For the toolpath generation, we created a triangular- or diamond-shaped pattern using polygonized isocurves. The precise tool orientation and position were deducted from the normal vectors of the base surface, considering the filament thickness and layer count. We investigated material spring-back tolerance by 3D scanning and compared the result with the simulation model.

**FIG. 5.**
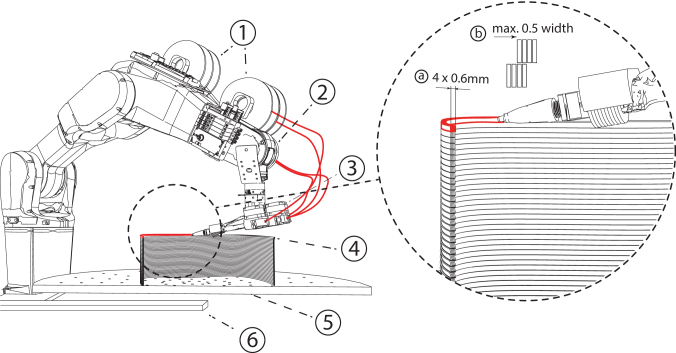
Robotic setup for multilayer lamination. Four filament strips from four spools (1) are printed on the robot (2), combined in the end effector (3), and laminated into the hot end into a square profile (4), to be able to print in a continuous AM process on a turntable (5) and a track (6). *Right*: Lamination schematic: Several filaments are combined and laminated into a square profile to be able to print in a continuous AM process, layer on layer through square profiles. a = combined square profile, b = maximum overhang-angle. AM, additive manufacturing.

For the fabrication of surfaces that are also curved in plan, a multilayer lamination technique was investigated. As a wicker strip has the highest flexibility in its flat surface direction, multiple filament strings were combined to join and create one composite and square extrusion (Fig. 5). This technique has the advantage of being able to stack the extrusion in layers, while being capable of applying strong curvatures. The extrusion can keep its applied structure due to the combined filaments' differential length in occurring curvatures. For combining the willow filaments, the adhesive is reactivated by running through a heated chamber inside the end effector.

**FIG. 6.**
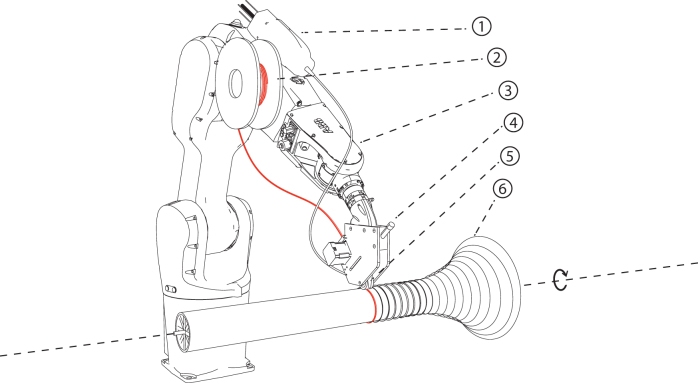
Robotic setup for hollow-core additive timber manufacturing: glue dispenser (1), filament spool (2), robot (3), end effector (4), UV-curing nozzle (5), and a revolving shape around a rotating axis (6).

For the fabrication of hollow profiles, filament strips can be applied using a rotating external axis. As molds, we used dissected structures made from plates cut to the specific profile geometries. In comparison with existing industrial winding technologies, gradual transformations between flat to hollow core profiles and freeform profiles were investigated (Fig. 6). We also investigated application patterns that minimize filament buckling. For variable geometries, the cross section and the pitch change gradually, and so, the speed and the angle of the robot or the rotation axis must be constantly adjusted. The toolpath for this typology is generated through the following steps. First, a surface of revolution is divided into sections along its length. Then the tangent at the start point of the second line is matched with the tangent at the end point of the last line using Newton's method, resulting in a locally geodesic line. The start angle can be adjusted to create lines with different slopes. The toolpath is derived by creating targets that are rotated according to the slope of the line. For each target, the robot speed must be calculated separately.

### Structural testing of samples

**FIG. 7.**
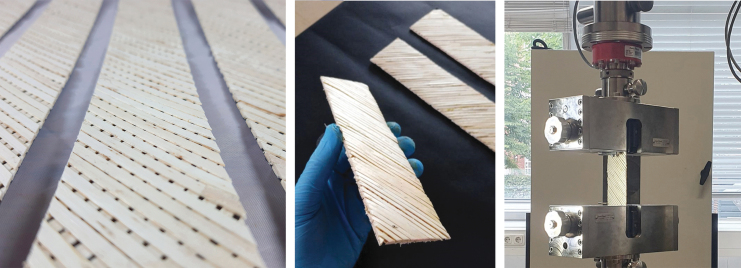
The panels for testing were, five at a time, cut in angles of 0°, 22.5°, 45°, 67.5°, and 90° from one textile-like printed panel. The tensile shear specimens were tested on a Zwick&Roll Z100 universal testing machine at a test speed of 10 mm/min.

To determine the material properties of the additively produced composite material, solid specimens with a dense filament layup were printed, and initial tensile tests were carried out. Repeating panels measuring 250 mm by 40 mm with varying filaments and therefore fiber orientations within two layers were printed as the first flat test bodies. The filament directions are oriented in a 90° angle to each other with a distance of 4.5 mm at the centerline or 1.5 mm between the filament outlines. Stress angles of 0°, 22.5°, 45°, 67.5°, and 90° were used as parameters for structural tension tests. Five test pieces of each angular variety were cut from a large printed plate, and tested (Fig. 7).

## Results

### Material development

With microsplitting and trimming, we were able to homogenize the willow strips to join them to a continuous filament for AM with a thickness of ∼0.3 mm and an adjustable width of 2–5 mm. Through this homogenization process, we obtained uniform dimensions and appearance, as well as a lower variation of strength values. Since slight damages in the surface of the wood are removed by the processing, and severely damaged wooden rails are sorted out, the homogenized rails even achieve a higher tensile strength on average. The goal of the filament joint was to have a connection that is at least as strong as the material itself. Furthermore, it is essential for the printing process, and also for the later use phase of the manufactured structure, that the elastic properties of the joint area are similar to the rest of the wood filament. In addition to the choice of a suitable adhesive, the geometry of the joint is of importance. With correct design and simple mechanical pretreatment, sufficiently high-strength values can be achieved even with simple joining geometries (e.g., overlap bonding). As the cross section decreases, the production of more complex joining geometries such as the shank becomes more difficult, which is why there is still potential for research in this area. We also performed a test series comparing the tensile strength of the willow filament with a beech sample of the same profile, resulting in an average of 167.61 MPa for the willow compared with an average of 57.11 MPa for the beech samples.

### Binding experiments

**FIG. 8.**
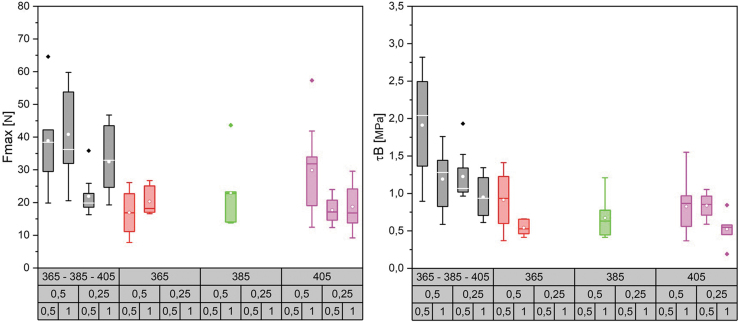
Maximum force (*left*) and adhesive strength (*right*) with use of acrylic resin and different parameter configurations. The legend on the *X*-axis describes on the first line the wavelengths used, followed by the irradiation time and the overlap length.

A parameter study was conducted, wherefore willow rails were joined overlapping in fiber directions (5 and 10 mm), using UV-activated acrylic resin with different wavelengths (365–405 nm) and irradiation times (0.25 and 0.5 s). If no boxplot graphic is given for a parameter configuration, less than 40% of the test specimens could be included in the evaluation. The graphs (Fig. 8) illustrate that the bond strength can be increased by combining different wavelengths, even with small joining zones and a short irradiation time. At the same time, it is evident that there is a relatively strong value dispersion and associated high standard deviation around the arithmetic mean value. The highest average strength 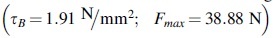 can be obtained for the combined irradiation with wavelengths 365/386/405 nm at an irradiation time of 0.5 s and an overlap length of 5 mm. Thirty percent of the samples could not be included in the evaluation due to premature substrate failure. By duplicating the overlap length to10 mm, this failure could be avoided. The average strength related to the bond area 
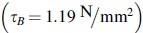
. The potentials of UV light-based joining of the wood filament can be seen clearly. To take maximum advantage of this potential, it is necessary to take a closer look at the binding process and consequently to improve the mechanical performance.

### Robotic AM

We investigated our AM technology for four different geometric typologies: flat components, curved surfaces, multilayer laminated curved walls, and hollow profiles. We also implemented a robotic end effector for automated filament layup, cutting, and adhesive activation (Fig. 3), which can be used for the fabrication of all tested typologies, despite the curved walls, for which we developed a specific end effector, capable of multilayer lamination.

For flat components, we fabricated a testing series to determine the exact material properties. The results for structural testing as described in the section “additive timber manufacturing”.

**FIG. 9.**
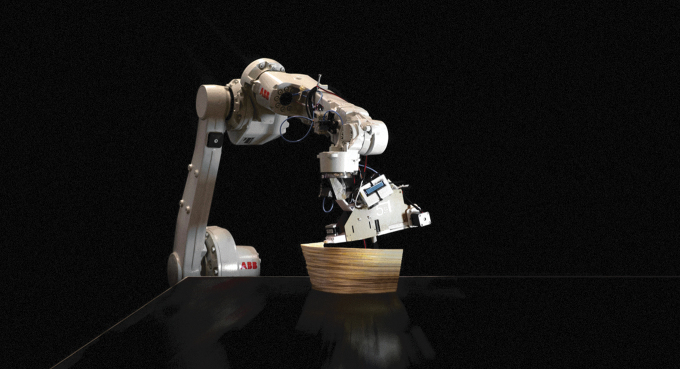
Printing result of the multilayer lamination technique. The flexibility of the willow material also enables curvature printing, as examined in Ref.^46^ We identified a minimum radius of 20 mm for in-plan curvature and a maximum overhang angle of 22.5° for the printing of larger parts of walls.

For multilayer laminated curved walls, a series of small-scale tests were effectuated for the in-place lamination of three to five filaments and their geometry (Fig. 9). We identified a minimum radius of 20 mm for in-plan curvature and a maximum overhang angle of 22.5° for the printing of larger parts of walls. Details on the methods and results of this process can be found in a separate study.^47^

**FIG. 10.**
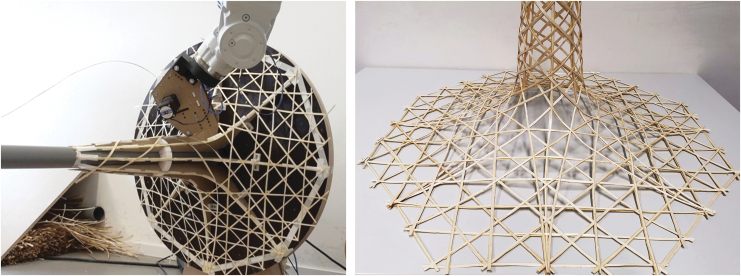
Result for robotic hollow-core additive timber manufacturing: transitions from flat components to supporting columns. The filament toolpath needs to be constructed from locally geodesic lines to prevent buckling and ensure efficient material distribution.

For hollow sections, we identified the following fabrication constraints, which are dependent on the flexibility of the willow filament and on the geometry and size of the end effector. In the direction of the filament application, there should be a minimum radius of 20 mm for the convex parts and 100 mm for the concave parts, perpendicular to the toolhead, a minim radius of 40 mm is required for concave parts (Fig. 3). In addition, the filament toolpath should be constructed from locally geodesic lines, to prevent buckling and ensure efficient material distribution. We fabricated several prototypes, which were also able to form transitions between flat, curved, and hollow sections (Fig. 10).

**FIG. 11.**
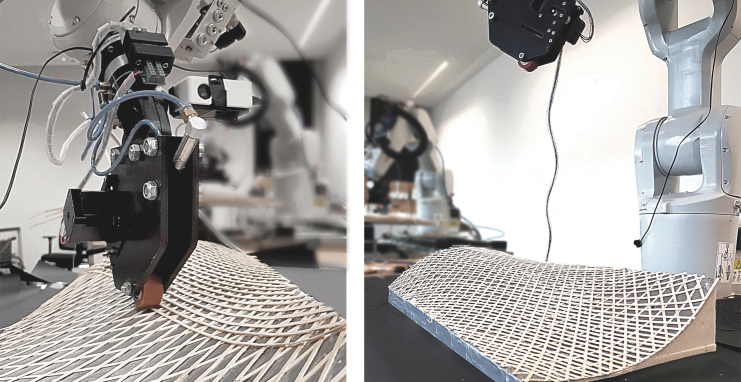
Curved surface additive timber manufacturing of a 590 × 271 mm three-layer pattern with a thickness-to-size ratio of *w* = 1/226, *l* = 1/492, by an industrial robot (Type ABB IRB 1200). We used a CNC milled foam as mold structure. CNC, Computerized Numerical Control.

**FIG. 12.**
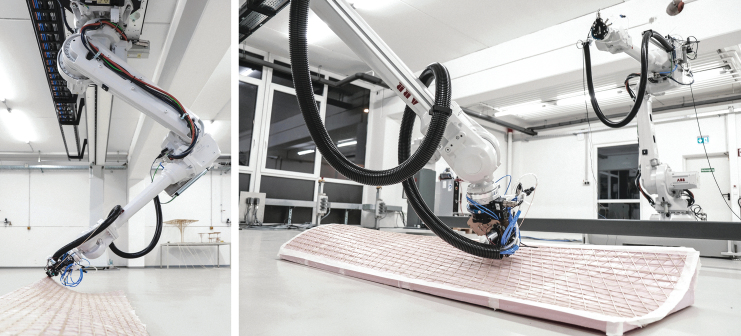
Curved surface additive timber manufacturing of a 2000 × 580 mm six-layer diamond pattern with a thickness-to-size ratio of *w* = 1/232, *l* = 1/800, by a hanging gantry robot IRB 4600-40/2.55. We used a CNC milled foam as mold structure. The printed part can be released from its support after finishing the print. CNC, Computerized Numerical Control.

**FIG. 13.**
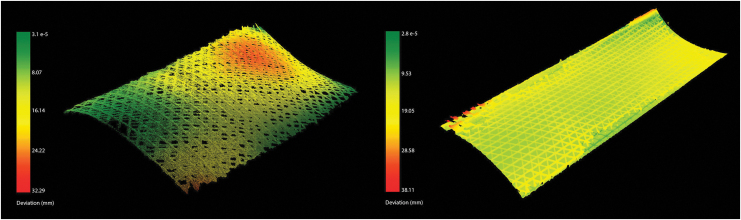
The divergence from the original surface was investigated through 3D scanning (colors are normalized), to evaluate how much the surfaces could maintain their geometry without any additional supports. *Left*: a 590 × 271 mm three-layer pattern. *Right*: a 2000 × 580 mm six-layer diamond pattern. Even though the thickness-to-size ratio for both samples is similar in the width, the larger one shows the effect of having a multiple layer lamination, with a much more even distribution of geometric divergence.

For curved surfaces, similar fabrication parameters resulted as for hollow sections. We fabricated a double curvature sample, and a change of positive to negative Gaussian curvature of 590 × 271 mm size with a three-layer diamond-shaped pattern, of around 1.2 mm thickness (Fig. 11), and a larger demonstrator (a slightly adapted and scaled version of the first sample) of 2000 × 580 mm size with six layers (around 2.5 mm thickness) and a total filament length of 174 m (Fig. 12). The geometry was chosen to evaluate the possibility of changing curvatures and also of twisting of the filament along its profile (around max. 25° of twisting compared with the surface normal). For the small pattern, we used our prototyping robot lab with IRB 1200s, and for the larger pattern we used a hanging gantry robot IRB 4600-40/2.55. The filament was applied to a polymer film coated with contact adhesive, which was wrapped around the previously milled foam to achieve good surface adhesion. Both were 3D scanned and compared with the original surface design (Fig. 13), to evaluate how much the surfaces could maintain their geometry without any additional supports. Even though the thickness-to-size ratio for both samples is similar in the width (small sample: *w* = 1/226, *l* = 1/492; large: *w* = 1/232, *l* = 1/800), the larger one shows the effect of having a multiple layer lamination, with a much more even distribution of geometric divergence.

### Structural testing

**FIG. 14.**
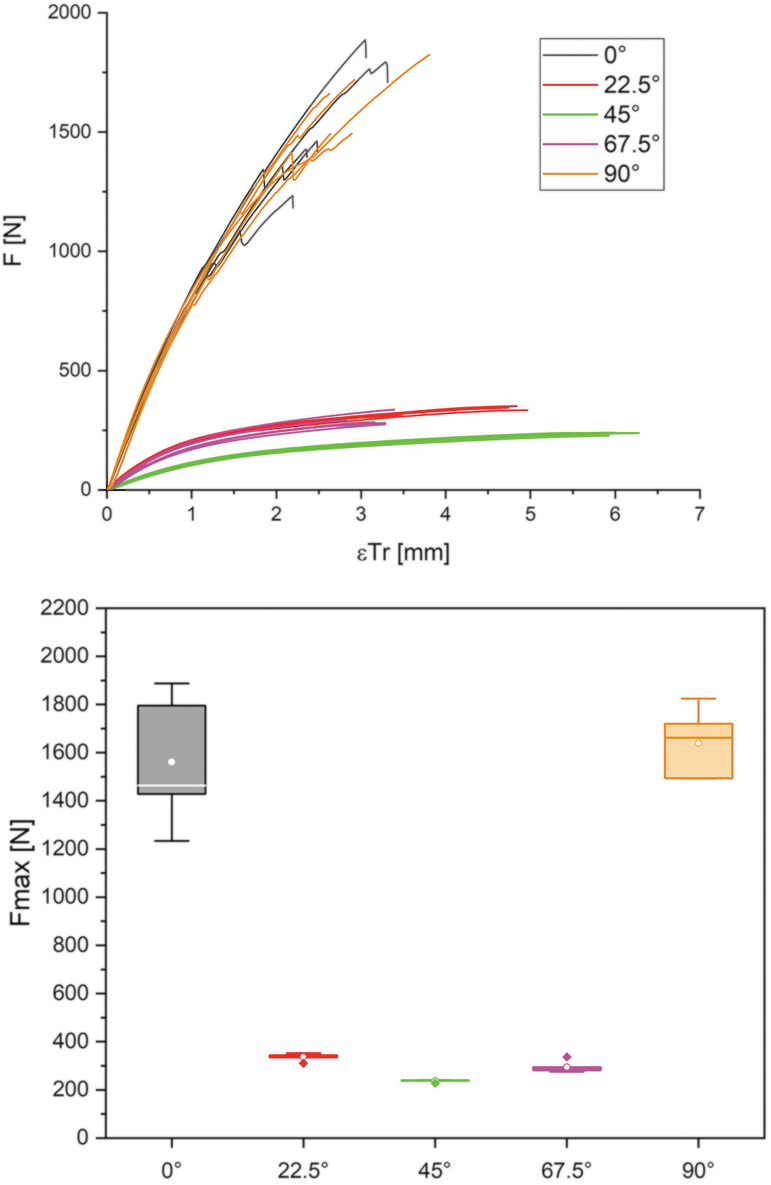
Force/displacement diagram (*left*) for the test specimens cut from the plates. Similar curve for specimens cut from the plates at angles of 0° and 90° or 22.5° and 67.5°. High fiber orientation in the loading direction gives the highest mechanical performance, while the 45° cut specimens show the highest elongation. The adjacent boxplot diagram (*right*) of the achievable maximum force supports the statements on the mechanical performance of different orientations of the layering. The primary force transmission via the wooden rails at 0° and 90° and the associated size influence factor cause a larger standard deviation in the values. The 22.5°/45°/67.5° variants show a lower mechanical performance in a narrow range of values.

To determine the material properties of the additive-produced composite material, tensile tests were conducted with the speed force values as in the Materials and Methods section. In addition to the force values, the traverse elongation was determined. Figure 14 shows on the left side a force/deformation curve and on the right side a boxplot diagram showing the values determined in the tensile test. The abovementioned plate cutouts are color-coded in both illustrations as follows: 0° black; 30° red; 60° green; 90° magenta; and 120° orange.

Looking at the force/deformation curve, the 0° and 90° test series stand out with very similar force/strain curves and the highest mechanical performance. The adjacent boxplot confirms this impression and certifies the 0° orientation an average strength of 1561 N with a standard deviation of 271 N (17.3%), while the 90° orientation has values of 1638 and 144 N (8.8%). For these two variants, the transmission of force is primarily via the wooden rails, which allows the best possible strength values to be reached. Since it is a natural material and at the same time a certain size influence factor must be taken into account, a higher standard deviation occurs in the values. The 22.5° and 67.5° orientation test specimens also show a similar course, through which the first mentioned ones deform more until material failure. On average, strengths of 336°N at a standard deviation of 15 N (4.5%) are achieved by these types of test specimens. Test specimens with 67.5° orientation fail on average at 294 N with a standard deviation of 24 N (8.2%). The lowest strength values are found in the test series of the 45° orientation, with an average of 237 N at 5 N standard deviation (2.1%). Compared with all other test series, these deform the most until failure occurs.

The Young's modulus $E_{t,0}$ was determined for each specimen individually based on the load/deformation curve. The calculation is based on DIN EN 408 by identifying the linear elastic area of the curve in a range from 10% to 40% of $F_{max}$. In this section, a linear regression analysis was carried out in each case, through which the correlation coefficient can be specified as >0.99 for all test specimens. For this reason, $E_{t,0}$ is given for each specimen based on the linear regression and the original range of the load/deformation curve. Table 1 summarizes the values for $E_{t,0}$ of the test series.

**Table 1.** Results for the Determination of the Modulus of Elasticity for the Plate Cutouts

Young's modulus (MPa)							
Orientation		N	Arithmetic mean	Standard deviation	Minimum	Median	Maximum
0°	Original	5	2797.89	130.54	2658.94	2781.90	2999.05
	Regression	5	2797.39	133.28	2654.17	2785.36	2999.10
22.5°	Original	5	824.86	25.64	792.82	818.77	863.58
	Regression	5	827.15	25.14	794.52	823.27	864.76
45°	Original	5	377.67	16.35	354.98	384.17	395.56
	Regression	5	379.12	15.99	356.40	384.59	396.77
77.5°	Original	5	725.88	50.93	682.33	713.75	812.26
	Regression	5	729.03	50.72	685.41	717.93	815.04
90°	Original	5	2641.40	96.22	2509.82	2635.42	2766.69
	Regression	5	2716.36	96.97	2577.95	2734.31	2835.57

The moduli are calculated based on the original load/deformation curve and linear regression (correlation coefficient >0.99). The arithmetic mean and the median are shown, together with the minimum and maximum values of each series.

The significantly higher strength values for the 0° and 90° cutouts are due to the large number of wooden rails oriented longitudinally to the load direction. A quasi unidirectional arrangement of the axial-anisotropic filament enables the comparatively high load absorption. The relatively strong scattering of the values around the average value can be explained by the number of lengthwise oriented fibers of the wood composite. The test specimens are cut out of solid boards and therefore it cannot be guaranteed that the same number of wood filaments is contained. Furthermore, the wood filament used is a naturally grown raw material. The material is mechanically processed, which gives it a certain homogeneity from an optical and mechanical point of view. However, with increasing path segment length, a certain size influencing factor can also come into play, through which natural and process-related defects are more likely to occur and can therefore cause a broader value spread.

## Discussion

Several AM technologies based on plastics, metals, and concrete are currently making the leap from prototype to series production at the scale needed for the construction industry. As one of the most digitized construction systems, wood-based processes combine the advantages in sustainability and seamless integration with existing computational workflows. Although wood construction is one of the most advanced building technologies in terms of digital design and prefabrication technologies, there are currently no large-scale AM techniques that are simultaneously able to continuously apply the material and use a wood-based material with an intact wood cell structure.

### Fabrication process

Our material and manufacturing process is the first technology that can apply natural wood with an intact fiber structure continuously in an additive robotic fabrication process with minimal adhesive use. Other techniques such as veneer-based lamination techniques exist in various scales, but they rely on discontinuous fabrication processes involving heavy and costly machinery. Currently known timber-based AM processes use wood in pulverized form, losing its inherent structural and mechanical properties. AM with natural fibers can be done continuously, but the fibers need to be embedded as a reinforcing filler in a polymer composite.

Both with contact and UV-adhesives, our material can be extruded at a multitude of the speed of traditional printers, potentially at robot speed, which will be subject to future research. This could be a substantial advantage to existing AM technologies, which are often uneconomical at large scale, due to their relatively slow fabrication speed. Our technology is aimed at architectural scale, where the printing resolution, geometric constraints of maximum curvature, and minimum bending radius of the filament are sufficient in most cases, while for small-scale prototyping, technologies with a higher resolution should be considered.

### Material capacity

For the research project, thin wooden splints were used, which, due to their dimensional stability, have a lower flexural slackness compared with single fibers, while at the same time having a high elasticity. By using wood as a natural fiber composite, it is also possible to achieve a high tensile strength in the direction of the fibers as well as a higher strength transverse to the direction of the fibers. A further essential advantage is the elimination of the costly extraction of the individual fibers, which in turn must be integrated into new composites or spun into a thread form. For a direct structural comparison of the compound material with veneer-based lamination, and filament- and fiber-based AM, further studies with the precise sample size are planned. Furthermore, we are currently investigating topology optimization techniques for linear filament structures, which can be used for material efficient structures. A comparison of a variety of patterns and their structural performance is subject for further studies, which could be used to tailor specific material behavior in different regions of a continuous surface.

### Sustainability

In comparison with veneer-based lamination, our technology results in a similar ecological footprint, if only the material values are considered, since both technologies use timber and adhesives. Due to the variability of application, less material can be potentially used to achieve similar structural and functional capacities. A substantial advantage is the fast harvesting possibilities of willow twigs. In comparison with construction timber, with a typical 35–70 years of growth before it can be harvested, willow twigs can be grown in short-rotation plantations, and harvested in cycles of 1–3 years.^48^ To produce wicker strips, primarily annual willow rods are used.^49^

In comparison with the extrusion of wood filaments in FDM printers, which currently only consist of a small proportion of grounded wood (up to 40%), our technology and compound additively manufactured material are made of solid material. Adhesive proportion is similar as in veneer-layered lumber (around 3–5%). A further reduction of the adhesive is planned through the investigation of microjetting application techniques.

In comparison with AM based on natural fibers, whose sourcing is complex and energy-consuming, the willow filament can be fabricated with simple and efficient techniques. Natural fibers have a much lower CO_2_ footprint than their carbon fiber counterparts, but there is still a huge difference to timber-based construction products. Compared with timber (Veneer-based: −424 kg CO_2_-eq, CLT: −524 kg CO_2_-eq (14)), this results in a largely positive CO_2_ footprint (350–975 kg CO_2_ per ton of fiber (34)), for the raw material even before its integration in a resin matrix.

## Conclusion

We proposed a novel material, a solid endless wood filament made from willow twigs, with a complete wood structure with continuous fibers, which through its high structural capacities could be used as a substitute for metal- and fiber-based products in the future, that is, membranes, interior fittings, façade components, furniture up to structural components. The filament can be fabricated from willow twigs, which are, compared with construction timber, extremely fast-growing locally harvested plants, resulting in substantial sustainability advantages. In this study, we characterized the material properties of the filament and the AM compound material, and investigated and compared suitable binding technologies. We developed AM technologies for four surface typologies and described their resulting geometric and fabrication constraints. We were able to fabricate a range of testing samples for flat, simply, and doubly curved samples and a large-scale demonstrator of a double-curved surface, showing the potential of AM in timber construction with solid wood materials. We compared the fabrication parameters, material capacities, and sustainability of the AM technology with veneer-based lamination techniques, existing wood filament 3D printing, and natural fiber-based AM. Strengthening the competitiveness of the bioeconomy is a logical consequence of a further implementation of this technology. In particular, our process offers a novel manufacturing process with entirely new possibilities for wood-based materials that can serve as substitute materials in architectural construction, furniture, and interior design, such as fiber composites. AM allows extremely efficient use of resources, and thus also targeted weight optimization and the realization of components with complex geometries, such as those required in automotive, caravan, and shipbuilding. In addition, acoustically effective elements can be produced by fine structuring and variation of the surface.
